# Implicit Learning of Recursive Context-Free Grammars

**DOI:** 10.1371/journal.pone.0045885

**Published:** 2012-10-19

**Authors:** Martin Rohrmeier, Qiufang Fu, Zoltan Dienes

**Affiliations:** 1 Cluster Languages of Emotion, Freie Universität Berlin, Berlin, Germany; 2 State Key Laboratory of Brain and Cognitive Science, Institute of Psychology, Chinese Academy of Sciences, Beijing, China; 3 Sackler Centre for Consciousness Science and School of Psychology, University of Sussex, Brighton, United Kingdom; Utrecht University, The Netherlands

## Abstract

Context-free grammars are fundamental for the description of linguistic syntax. However, most artificial grammar learning experiments have explored learning of simpler finite-state grammars, while studies exploring context-free grammars have not assessed awareness and implicitness. This paper explores the implicit learning of context-free grammars employing features of hierarchical organization, recursive embedding and long-distance dependencies. The grammars also featured the distinction between left- and right-branching structures, as well as between centre- and tail-embedding, both distinctions found in natural languages. People acquired unconscious knowledge of relations between grammatical classes even for dependencies over long distances, in ways that went beyond learning simpler relations (e.g. n-grams) between individual words. The structural distinctions drawn from linguistics also proved important as performance was greater for tail-embedding than centre-embedding structures. The results suggest the plausibility of implicit learning of complex context-free structures, which model some features of natural languages. They support the relevance of artificial grammar learning for probing mechanisms of language learning and challenge existing theories and computational models of implicit learning.

## Introduction

Humans seem to possess a remarkable facility to grasp new structures from the environment and generalise the use of this knowledge to other stimuli and domains [1]. We are able to master complex everyday activities such as steering a car along bends and in traffic [2], intercepting thrown objects [3], playing sports [4], hearing words in continuous speech [5], or improvising music in an ensemble [6]–[9] without full awareness of the knowledge enabling such activities. Similarly, native speakers of a language are able to understand and produce sentences without being able to fully articulate the grammatical rules they are applying. Children acquire and use grammatical knowledge from mere exposure or interaction with very little explicit input or teaching [10]. First and second language acquisition thus constitutes a prototypical case and one main example for implicit learning (e.g. [11]–[15]).

Most artificial grammar learning studies apply finite-state grammars, using letters, syllable sequences, tones, melodies, timbres, or visual symbols as terminals, producing strong evidence for implicit learning [16]–[24]. Similarly, numerous studies have investigated the acquisition of linguistic phrase structure using finite state grammars [25]–[32]. In the same context many studies have found that adults and children are able to learn different features of language based on their statistical properties and features such as word segmentation and word categories, without needing to refer to phrase structure grammar per se [5], [33]–[40]. Such research raises the question of what needs to be shown to demonstrate the implicit acquisition of linguistic syntax. Are finite state grammars and statistical learning sufficient to account for human implicit learning and language learning? However, as argued by [41]–[42], finite state grammars are not sufficiently expressive to capture linguistic syntactic recursion and the modularity and hierarchical organisation of constituents and phrases. The complexity of at least context-free grammars is required to capture these features (see e.g. [41], [43]–[46] for a discussion). To our knowledge, only a few studies have explored implicit learning beyond finite-state complexity (see below, [47]–[51]).

The purpose of this study is to investigate implicit learning of a linguistic context-free grammar above finite-state grammar complexity. The structure of the sequences produced by the grammars used in the study embody distinctive features of recursion [52], in particular, nested or tail recursion (see below), and hence involve hierarchical organisation and long-distance dependencies (note that we use the notion of hierarchical structure in the sense of *hierarchically nested dependency* relationships according with the definitions of recursion in [53] and the distinctions between formal languages drawn by [54]). In this context the study links to the recent debates about the learnability of recursive, centre-embedded structures in the cognitive sciences (see below).

## Background

One central aspect of language syntax concerns the organisation of words, constituents and phrases in nested, recursive ways [43], [55]. An example would be “the old garden at the rear of the house” which acts as a noun phrase like the single “the garden”; as a noun phrase both could fit the context “… is beautiful”. Similarly, the words in the sentence “the Labrador which chased the poodle that was hiding barked” fall into recursively dependent constituents: the Labrador [which chased the poodle [that was hiding]] barked. The understanding of a sentence like the above requires the correct parse of the syntactic and semantic dependencies to reconstruct the appropriate sentence meaning (which of the two dogs barked?). Generally, however, parsing of semantic and syntactic dependencies interact (see also [56]).

Another example of a recursive German sentence would be: “Hans sagte, dass Peter Maria dem Mann den Zaun streichen helfen sah.” (“Hans said that Peter saw Maria help the man paint the fence.”). Embedded relative clauses like the English or German examples involve recursive nested hierarchical embedding and nonadjacent dependencies (e.g. “the Labrador …. barked”, “Maria … helfen”). In the English example there are two instances of nested tail-recursion (each embedded sequence adjacent to the end of a sequence, e.g. “the poodle [that was hiding]”; we henceforth refer to embedded structures generated by tail-recursion as “tail-embedding”) and one instance of centre-embedding (“the Labrador […] barked”). In contrast the German sentence features three instances of recursive nested centre-embedding (“[Peter [Maria [dem Mann … streichen] helfen] sah]”). The fact that the dependencies in either language are generated recursively entails that they are potentially unbounded and infinite in the sense that there is no theoretical upper limit for the number of tail- or centre-embedded structures if the pattern would be continued (not considering limitations of performance such as working memory). These potentially unbounded nested dependencies constitute the core of the argument for recursion at the heart of the human language faculty ([41], cf [44]–[46], [57]–[58]).

Finite-state grammars can express simple forms of tail-recursion and limited nonadjacent dependencies. This constitutes a difference between finite-state grammars (as defined by rewrite rules that only add elements to one side) and Markov models (i.e. as represented by a table of transition probabilities). While they largely overlap, they are different [41]. demonstrates that a finite-state grammar (in notable contrast to n-th order Markov or n-gram models) can express an unbounded nonlocal dependency using tail recursion (e.g. AX*B | CX*D). In words, the expression means: a set of sequences in which either A is followed by any number of X and B or C followed by any number of X and D. Hence an initial A implies B after any number of X, and the same for C and D. Therefore any Markov or n-gram model of finite length will not be able to express this (unlimited) nonlocal dependency although it easily be constituted by a simple finite-state grammar.

Context-free grammars in contrast can express forms of nested dependencies that can be proven not to be finite-state. Unbounded recursively nested dependencies like UA^n^VB^n^W (where U, V, W may be any sequence of terminal events or empty) can be expressed by context-free, but not finite-state grammars. The German example above constitutes a sentence that exhibits this type of dependency “Peter_1_ Maria_2_ dem Mann_3_ streichen_3_ helfen_2_ sah_1_”.

Research exploring learning or processing of recursion and context-free grammars bears one particular caveat: The difference between context-free and finite-state grammars relates to potentially unbounded dependencies while, trivially, a finite set of sequences can be expressed by an all encompassing finite-state grammar. However, an unlimited number of dependencies cannot be explored experimentally. On the other hand, finite-state grammars expressing nonadjacent dependencies or finite examples of context-free sequences are redundant: for instance, AX*B | CX*D encodes the identical intermitting X* twice, and a finite-state grammar encoding general nonlocal dependencies between *n* pairs of symbols has to represent the intermitting sequences *n* times (second or third order embeddings would accordingly let the number of multiple representations grow exponentially). Thus, although it may be possible to express such bounded structures by a finite-state grammar, a context-free grammar achieves a more parsimonious representation. Sometimes the simpler psychological explanation for what has been learned will involve a grammar higher up the Chomsky hierarchy.

Various research has been performed in this line of research linking the field of implicit learning with syntax acquisition and recursion (cf [59]–[60]). In order to put our study in context we systematically review existing research on (not necessarily implicit) learning of recursion, nonadjacent dependencies and word classes.

### Recursion and context-free structure

The exploration of the learning of realistic features of context-free grammars is linked with one current cognitive debate concerning the processing and learnability of recursive structures. Recursion is argued to be situated at the heart of the human faculty of language (e.g. [54], [57], [61]. Hierarchically nested structures and recursion in various forms of human communication, such as language, music [62]–[66] as well as planned action have been argued to be unique to human cognition [48]–[49], [67]. [67]–[68] situates the hierarchical organisation of language and music within a broader human capacity of recursion, a position that is similarly argued by [69]. In this context, the question of how humans form, acquire and manage complex recursively embedded hierarchical structures constitutes a core question in the area. Again, in line with [53], hierarchical organisation entails the representation of dependency relationships between constituents (at multiple levels but not necessarily based on the same principles or rules). Recursive embedding entails the representation of dependency relationships based on the same rule or principle; the recursive nature of the embedding step further entails that the resulting hierarchical organisation generalises to levels of embedding that are potentially unbounded and may not be observed.

Context-free grammars, or phrase-structure grammars, constitute the simplest form of grammars to embody features of unbounded nested embeddings in the Chomsky hierarchy [41], [70]. The Chomsky hierarchy characterises four types of formal languages [70]–[71] of increasing complexity: regular or finite state languages, context-free languages, context-sensitive languages and recursively enumerable languages. The types of grammar which produce these languages differ by systematic steps of generalisation of the form of the rewrite rules. Whereas finite-state grammar rules embody the most restrictions, the top level (type-0) rules are entirely unrestricted. By virtue of dropping restrictions, every more complex grammar and language becomes a superset of the less complex grammar or language. Accordingly, context-free grammars include all finite-state grammars, and context-sensitive grammars include all context-free grammars and finite state grammars. Thus, there are grammars employing context-free rules which are in fact expressible by finite-state grammars. The core differences between finite-state and context-free languages lie in the features of recursive, centre-embedded structures [71].

The current empirical evidence about the learning and perception of hierarchical recursive structures is ambiguous, and, as a result, discussion in the area is ongoing. Several studies employed very simple grammars of the type A^n^B^n^ and variants of it: [48] argued to have found evidence for learning of simple regular (finite-state) and nonregular structures (AB)^n^ vs. A^n^B^n^ in two species. [72]–[75], using similar methodology, found two different brain regions are associated with the acquisition of finite-state and context-free grammars. In contrast [76], did not find that participants were able to acquire specific features of the grammar used by Fitch and Hauser. Similarly [77], argued that Fitch and Hauser's original experiments contained a methodological flaw and found people could not learn the grammar A_1_A_2_A_3_B_3_B_2_B_1_ which forced hierarchical embedding for its recognition under incidental learning conditions (the grammar A^n^B^n^ could be simply distinguished based on mere word class counting). Similarly [78] argued that participants using the Friederici et al material engaged explicitly in counting strategies and did not learn the hierarchical structure per se. Thus, the simplistic and reduced case of an A^n^B^n^ language may not provide sufficient context and grammatical complexity for people to generalise a genuine context-free grammar. In a recent study, however [79], (see also [80]) argued that sufficient exposure to exemplars without embedding (zero level embedding) and staged input may explain found differences regarding the learnability of A^n^B^n^ grammars [81]. argued that an increase in complexity could help rather than hinder people to learn grammatical structures. This provides the motivation why the present study adopted more complex context-free grammatical structures which employed more features of a natural syntax as materials.

In an impressive study [50], trained subjects for 30 minutes on letter strings instantiating crossed or nested dependencies (as indexed variants of A^n^B^n^) on each of nine days. People could discriminate grammatical from non-grammatical strings after this extensive training, yet could not say which letters were paired as dependents. While their results may be due to people unconsciously learning hierarchical structure, there remains a confound in their materials. We know already that people learn the repetition structures of letter strings, i.e. in which positions of a string letters are repeats of which other positions [82]–[84]. Grammaticality was completely confounded with repetition structure, and if people had consciously learned repetition structure, it would explain people's classification performance and poor verbal report of the hierarchical dependencies. Thus, the issue of whether people can unconsciously learn hierarchical structure remains open.

As discussed above, there are several other studies which employ sequences which are produced from context-free grammars: the structures used by [72] and by [34], [85]. Whereas the above A^n^B^n^ structures were proved to be irreducibly context-free, the grammars used by [34] and [85] can be expressed by a finite-state grammar. For instance [31], showed a finite-state representation of the grammar they used in several studies (called BROCANTO) and a similar step could be done for the studies by Saffran [34], [85] (see Appendix S1). Accordingly, although Saffran's grammars and BROCANTO incorporate features of realistic sequential linguistic word order, they do not incorporate the prototypical context-free features of nested centre-embedding and multiple (potentially unbounded) long-distance dependencies that are required for context-free grammar complexity and that characterise one distinctive feature of human linguistic structures. Moreover, most of the studies relevant to learning context free grammars do not integrate measures of awareness to investigate the extent to which the acquired knowledge is implicit (unconscious) or explicit (conscious) into their methodology [15]. Further, only a few studies relating to second language acquisition employ conditions known to be conducive to implicit learning (for good examples see [13], [86]–[91].

### Learning long-distance dependencies

A feature that is closely related to the above debate is nonadjacent dependencies, as centre-embedding context free grammars imply long distance dependencies [14]. pointed out that nonadjacent dependencies have not been sufficiently explored yet from a statistical or implicit learning perspective. Using letters as stimuli, people can learn repetition patterns across stimuli [84], [92], a simple form of nonadjacent dependency. However, under the standard conditions used in artificial grammar learning studies, people do not implicitly learn nonlocal distance associations between letters which are not repeats (in the biconditional grammars of [93], and [94]. [95] also did not find evidence of learning of nonadjacent dependencies in syllable sequences. However, they found adults could acquire long-distance dependency relationships, only between literally nonadjacent vowels (or consonants) which actually constituted successive vowels (or consonants). [96] found learning of nonadjacent dependencies in tone sequences only when the relevant structures were separated from the surrounding structures by auditory streaming. Consistently [97], also found that nonadjacent dependencies between non-musical noises could be learnt only when perceptual similarity cues were introduced.

In sum, it has been difficult to find learning of long distance dependencies in the lab when simple perceptual cues did not direct attention to corresponding elements. Such research does not bode well for finding implicit learning of phrase structure in the lab, as the long distance dependencies in the research just reviewed were not even as complex as those instantiating phrase structure grammar. However, when dependencies have been put into a context of more structure, long distance dependencies have been learned in the lab. [98] found leaning across an intervening element when the intervening element was variable. [47] and [99] found that when the long distance dependencies were structured (namely, by forming a musical inversion, retrograde or transpose, which cannot be expressed through finite state grammars), they were learned (see also [51], [100], [101]). Perhaps placing long distance dependencies in certain ecological context-free structures actually helps learning.

### Implicit learning of word classes

Learning a natural phrase-structure grammar involves not only the learning of the syntactic dependency structure, but also distinguishing terminal elements (i.e. the elements forming the sequence: words in a sentence, notes in a melody, etc) from grammatical classes and acquiring knowledge about the relationships between terminals and grammatical class. For example, when learning English, one needs to infer which word class (i.e. noun, verb, adjective etc) each word (the terminals) belongs to, and the relationship between the word classes. Several studies have explored learning of word classes. A few studies have investigated the learning of classes in the artificial grammar learning paradigm. For example [102], applied a simple finite state grammar (Q)AXB(B) in which any of the categories were realised by two or three words each. They found that participants trained on sequences from that system were able to generalise to new (unseen) strings that conformed to the abstract classes. However, the study did not test for awareness or implicitness of the acquired knowledge. One of our aims will be to explore whether relations between grammatical classes can be implicitly learned.

### Motivation

To explore whether people can *unconsciously* learn context-free structures that are more advanced than A^n^B^n^ and reflect some natural linguistic patterns (following [81], as above), the present study adopts simplified linguistic context-free grammars, which generate recursive, centre-embedded structures. The artificial context-free grammars were designed to resemble some natural linguistic structures in an abstract way and also to feature a set of different word classes and terminals. To condense the discussed linguistic features into a small set of artificial grammar rules, we chose grammars similar to [34], [85]. However, the aim of this study was to model specifically embedded structures such as “The dog [who chased the cat [that was hiding]] barked”. The surface structures were chosen to be sentences of monosyllabic words in the auditory domain to correspond roughly to ecological listening conditions.

A key difference between phrase-structure grammars is whether they are right branching (as in English) or left branching (as in Chinese). Thus, we will use two variants of a grammar, i.e. a right branching grammar and a left branching grammar, to explore the relevance of this distinction for adult implicit learning. In addition, we will have two further variants of each of these grammars, reflecting another distinction between natural language grammars [103], [104], namely centre-embedding or tail-embedding (or nested recursion vs. tail recursion). The structures we are using will feature up to three levels of embedding, which we refer to henceforth as “layer” 1,2 and 3. Their difference amounts to whether or not the third layer is centre or tail embedded in the second. For example, consider the English sentence “The dog, [who chased the cat, [who caught the mouse]], barked”. “The dog barked” would be the first layer; “who chased the cat” would be the second, and “who caught the mouse” would be the third. Note the third relative clause is tail-embedded and hence adjacent to the second (in terms of word order) rather than being centre-embedded. Now consider its German equivalent “Der Hund, [der die Katze, [die die Maus fing], jagte], bellte”(transliteration: “The dog, [who the cat, [who the mouse caught], chased], barked”), where the third relative clause is embedded in the middle of the second. From a cognitive perspective we would predict that the word order of the simpler former (adjacent) case would be easier to learn than the latter (centre-embedded) structure. With respect to our grammars, we will refers to this difference as “tail-embedding” versus “centre embedding” grammars.

The following grammar was chosen as the tail embedding, right branching grammar (in analogy with English):

1. S→NP VP
2. VP→V_1_ | V_2_ NP
3. NP→N | N CP
4. CP→R VP

The rules of this abstract grammar intend to model simple linguistic relationships: it describes main sentence (S), consisting of a noun phrase (NP) and a verbal phrase (VP), and a simple complementiser phrase (CP). The grammar contains three classes of words that we have glossed as verbs (V), nouns (N) and a relative-clause marker/relative pronoun (R), which model the corresponding natural language classes in an abstract way. Here, S, VP, NP, and CP denote nonterminals and V, N, R denote terminals. Rule 1 indicates that the sentence consists of a noun phrase and a verbal phrase. Rule 2 indicates a verbs can be combined with an additional noun (modelling a distinction similar to transitive or intransitive verbs). The rule distinguishes verbs that entail another NP (V_2_) or verbs that do not (V_1_). Rule 3 indicates that noun phrases can consist of a single noun or a noun with a complementiser phrase attached. Rule 4 indicates that a complementiser phrase is made by a verbal phrase and a marker R which creates the potential of recursive generation, as it enables a VP to recursively be attached to an NP. This optional recursive production ensures that the production process terminates. Thus, the grammatical rules are similar to realistic structures in an abstract way.

**Figure 1:**
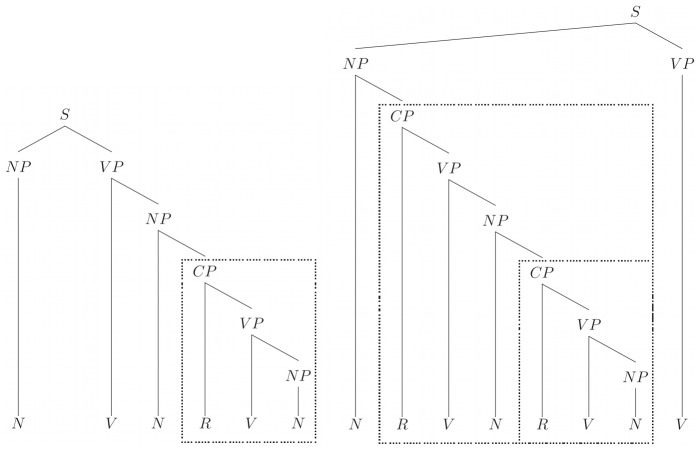
Right-branching grammatical structure trees allowed by the tail-embedding right branching grammar. Each subordinate CP corresponds with an embedded layer ( e.g. layer 2 on the left tree, and layer 2 & 3 on the right tree). Note that there is no centre-embedding on the left tree, while there is centre-embedding in the right tree with respect to the top NV structure).

When rules (4) and (2) are used to rewrite rule (3), there are three forms of NP with increasing complexity: NP→N | N R V | N [R V NP]. The third form shows clearly the centre-embedding of the relative clause with respect to the main clause (only). This structure is exemplified in the sequence “[The dog [who chased the cat [that was hiding]] barked]”. The sentence is made by an NP “The dog who chased [the cat that was hiding]” with the structure of “N [R V N [R V]]” and a VP which is made by a V “barked”. Figure 1 displays two different sequences created by the grammar. The tree structure illustrates how non-adjacent dependencies are produced in this grammar, and how the structure “R V (N)” is generated. The grammar creates right-branching dependencies as the relative clause “R V N” is joined to the right of a noun (like “The boy who kissed the girl”).

For the tail embedding left-branching condition, the corresponding rules are:

1. S→NP VP_1_
2. VP_1_→V_1_ | V_2_ NP
3. VP_2_→V_1_ | NP V_2_
4. NP→N | CP N
5. CP→VP_2_ R

Now consider the centre embedding grammars. For the right-branching grammar (in analogy with embedding structures in German), the corresponding rules are:

S→NP VP_1_
(2a) VP_1_→V_1_ | V_2_ N
(2b) VP_2_→V_1_ | NP V_2_
NP→N | N CP
CP→R VP_2_

To generate centre embedding left-branching grammatical structures (in analogy with embedding structures in Chinese) the corresponding rules are:

1. S→NP VP
2. VP→V_1_ | V_2_ NP
3. NP→N | CP N
4. CP→VP R

**Figure 2:**
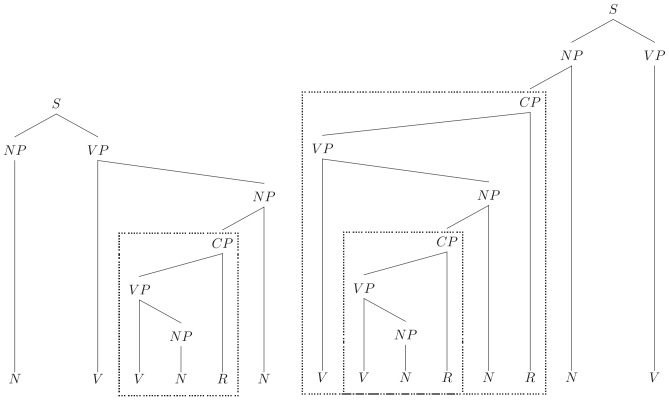
Left-branching grammatical structure trees allowed by the centre-embedding left branching grammar. (note that the subordinate CP embedding creates a nonlocal dependencies on the superordinate level).

Figure 2 displays two different sequences created by the centre-embedding left-branching grammar. The tree structure illustrates the way in which nonlocal dependencies are produced in the left-branching grammar, and how the structure “V NP R” is recursively embedded. To generate the final surface sentences, each of the terminal symbols V, N, R in each abstract structure was randomly replaced by one of a set of corresponding monosyllabic words for each class.

As outlined above, the purpose of the experiment was to use these four different artificial grammars to investigate whether people can become unconsciously sensitive to different types of recursive context-free grammars.To explore whether people can be sensitive to violations of the nested recursive structure, two thirds of the ungrammatical structures were designed violating only one embedded structure with the other levels remaining grammatically intact. Accordingly, a violation may span across an embedding. Sensitivity to such a violation would provide prima facie evidence of learning long-distance dependencies created by a recursive hierarchical grammar. However, we already know from past research that people are sensitive to bigram and trigram frequencies [105]–[107] and to repetition structure [82]–[84]. Thus, we statistically control these variables (contrast e.g. [50], [108]; further, a preliminary computational analysis suggested that it was not possible to balance grammatical and ungrammatical stimuli for indistinguishable bi- and trigram frequencies). The variables will be controlled at both the level of terminals (e.g. the actual word bigrams people were exposed to) and classes (e.g. the sequence noun-verb is a particular bigram). Implicit sensitivity to class-level n-grams and repetitions independent of terminal-level n-grams and repetition is itself an interesting independent question important for implicit learning research. In this context the present experiment contributes to research on the limits of what can be learned implicitly, as well as the role of implicit learning in first and second language learning (cf [13]–[15]). In addition, secondary questions concerned whether branching type and grammatical complexity would influence the acquisition of the phrase structures and whether participants' native language (Chinese) would affect the proficiency of learning of the type of grammar. The centre embedding left-branching structures were consistent with the grammatical structures of participants' native language while the right-branching and tail embedding structures were not. On the other hand, finding that people can incidentally and implicitly learn context-free grammars will be an interesting challenge for computational models of implicit learning (cf [109]), since they predominantly tend to be good at learning chunks and associations [110].

We are interested in the structures that can be learnt *implicitly* or *unconsciously*. The fact that people learn programming languages intentionally and consciously means there is little novelty in showing people can *consciously* learn artificial context-free grammars. People manifestly do this every day. However, whether structures more complex than chunks, and in this case produced by context-free grammars, can be learned *implicitly* by adults remains an important open question. For this purpose we chose to employ the Process Dissociation Procedure (cf [111]) as well as additional confidence judgments for assessing the conscious status of the acquired knowledge.

## Materials and Methods

### Participants

We recruited four groups to be trained on either tail or centre embedding structures and either left-branching or right-branching grammatical structures in the training phase. Correspondingly, we recruited four control groups for these conditions. One hundred and sixty-one undergraduate students (77 male, 84 female) in Beijing participated in the experiment. The mean age of the group was 22.3 years. The participants were randomly assigned to experimental or control groups for one of the four conditions combining tail or centre embedding left-branching and right-branching (*n* = 20 or 21 for each condition). Each participant was paid a 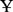
20 attendance fee (about three US dollars). The experimental protocol was approved by the institutional review board of the Institute of Psychology, Chinese Academy of Sciences, China. All participants provided informed consent prior to the experiment.

### Materials

#### Stimulus structures

With a maximum number of three embeddings, each of the four grammars produced 18 different abstract structures with a length from two to nine words. To generate the final surface sentences, each of the terminal symbols V, N, R in each abstract structure was subsequently replaced by one of a set of corresponding monosyllabic words for each class. There were four words for the V class, four words for the N class, and one word for the R class. An exhaustive recursive enumeration of all possible surface structures created a pool of several thousand different terminal sequences for each of the grammars.

To assess participants' ability to recognise *abstract* grammatical structures (order of word classes) independently of whether they belonged to the training set, we divided the abstract grammatical structures into *old-grammatical* structures which were presented in both training and test phases and *new-grammatical* structures which were presented only in the testing phase. The old and new grammatical structures each featured five 2-layer structures and three 3-layer structures. The two 1-layer structures were assigned purely to the old-grammatical set because of the small number of structure types. The 2- and 3-layer structures were randomly and equally assigned to old- and new-grammatical structures.

The training set consisted of 168 different sentences which included 16 1-layer structures (i.e. two old-grammatical structures instantiated randomly with terminals in eight different ways each), 80 2-layer structures (i.e. five 2-layer old grammatical structures instantiated randomly with terminals in 16 different ways) and 72 3-layer structures (i.e. three 3-layer old grammatical structures instantiated randomly with terminals in 24 different ways). The 2- and 3-layer structures were repeated two and three times because they are plausibly more difficult to learn than 1-layer structures.

In order to assess the acquisition of structural knowledge, we constructed two kinds of ungrammatical structures: layer-ungrammatical structures which violated only one layer of the grammatical structures and random-ungrammatical structures. The length of ungrammatical stimuli always matched that of the corresponding grammatical structure. There were 22 random-ungrammatical structures which matched six 1-layer structures (i.e. the two 1-layer structures repeated twice), ten 2-layer structures and six 3-layer structures (i.e. each abstract 2-layer and 3-layer structure repeated once). There were 44 layer-violating structures which featured the systematic violation of one of the embedded layers in each grammar. For instance, if the grammar belongs to the centre embedding left-branching, its first layer would be either “N V N” or “N V” and its second and third layer would be either “V R” or “V N R”. The violation of the first layer of “N V N” was either “V N N” or “N R V”; similarly,“N V” became either “R V” or “V N”. Thus, a grammatical structure “N V (V N R) N”, in which the first layer “N V N” is to be violated, became either “V N (V N R) N” or “N R (V N R) V”. Similarly, the violation of a second or third layer of “V N R” was either “V V R” or “N R V”; and “V R” became either “R V” or “N R”. Thus, a grammatical structure “N V (V N R) N”, in which the second layer “V N R” was to be violated, became either “N V (V V R) N” or “N V (N R V) N”. Accordingly, the violations of the layer 1 and layer 2 could induce long-distance (nonlocal) dependencies when there was (correct) centre-embedding because the superordinate ungrammatical layer would be intermitted. Altogether the layer-violating structures for each condition included six 1-layer structures (i.e. the two 1-layer structures violated in their one layer in different ways), 20 2-layer structures (i.e. ten 2-layer structures violated in their first or second layer, respectively) and 18 3-layer structures (i.e. six 3-layer structures violated in their first, second and third layer, respectively). Appendix S2 lists the stimulus sequences used for training and testing.

#### Stimulus rendering

In analogy to the paradigms by Saffran et al., as well as for the sake of simplicity, monosyllabic words were used as terminals. All terminal monosyllabic words were recorded from a professional Chinese Native speaker. The words used were “wao”, “yai”, “piu”, “shin”, “bam”, “fai”, “ti”, “ra”, “ki”. These phonemes/words were pronounced without tone (words were pronounced without tone in order to make a future experiment with Western participants possible). The combination of phonemes in a syllabic word violated Chinese rules for sequencing. Hence the words were not meaningful, in Chinese. Four words were randomly chosen for the V class, four words for the N class, and one word for the R class. For the construction of the stimulus sentences, the monosyllabic words were computationally concatenated to the respective auditory sequences using CSOUND. The sequences were automatically concatenated in order to avoid speaker produced intonation patterns, timing, etc. (cf. [112] for a detailed formal analysis of potential interactions between intonational effects with parsing). The CSOUND score files which specified the respective order of syllables were created using a MATLAB script that converted the randomly chosen set of terminal sequence structures into CSOUND score file format.

### Procedure

The experiment was run using a Flash-environment. There were two phases in the experimental procedure: a training phase and a testing phase.

#### Training phase

Participants were exposed to the set of 168 training stimuli under incidental learning conditions using a word counting distractor task. Before participants gave their word count, the sentence could be repeated as often as the participant wanted. The possibility of repeating stimuli according to the participant is analogous to one standard method in artificial grammar learning to let participants repeat stimuli (e.g. letter sequences) until they could recall them correctly (e.g., [1]). All training sentences were randomly divided into 8 blocks; each block included 21 sequences. There was an interval of at least 30 seconds between any two blocks. For the experimental group, the sentences were all old-grammatical structures. For the control group, the sentences were all random-ungrammatical structures.

#### Testing phase

Both experimental and control groups received identical instructions throughout the entire experiment. Following the Process Dissociation Procedure (PDP), the testing phase involved two tests: an inclusion and an exclusion test (While conscious and unconscious knowledge both contribute to picking the familiar item in inclusion performance, they conflict in the exclusion condition (as explicit knowledge would lead the participant to choose the unfamiliar item). Hence unconscious but not conscious knowledge of the grammaticality of the item could lead to the grammatical item nonetheless being chosen in exclusion; the difference between inclusion and exclusion performance makes it possible to estimate the amount of conscious knowledge, cf [111], [113]–[114]). In the inclusion test, participants listened to 66 pairs of sentences. Each trial pair featured one grammatical and one ungrammatical sentence. Participants were asked to choose the one that sounded familiar to them with respect to whether it appeared in the training phase. Subsequently participants specified their level of confidence on a scale from 50% to 100%, where 50% meant completely guessing, and 100% meant absolutely certain (i.e. to give a confidence rating; see [115]–[117], for discussion of such subjective measures of awareness). The subsequent exclusion test was carried out precisely as the inclusion test, except that participants were asked to pick the one sentence which sounded unfamiliar to them. Following the methodological conclusions by [118] the exclusion test always followed the inclusion test ([119] found that the order did not affect performance). There were 132 sequences in each test for both the experimental and control groups. As outlined above, half of the sequences were ungrammatical including 22 random-ungrammatical and 44 layer-ungrammatical structures; half were grammatical including 39 old-grammatical and 27 new-grammatical structures. Each grammatical structure matched a corresponding ungrammatical structure in length. The stimulus pairs appeared in a different random order for each participant.

Finally, participants were given a category identification test. They were told that the words in the training contained words from different categories such as nouns or verbs. They were then given 14 trials, in each trial they were presented with three words (two belonging to the same category) and were asked to try their best to choose two out of the three words which belonged to the same category.

## Results

We will consider the following questions in order: what have people learned, as shown by what violations they can detect? In particular, can people learn long distance dependencies? Is such knowledge modulated by the type of grammar (left vs. right branching, tail versus centre embedding)? Is the knowledge conscious or unconscious? And if we control for chunking and repetition structure, are people still sensitive to the long distance dependencies inherent in the grammars? Finally, can people classify the words that belong to one class? We focus the results section on these key questions.

### What was learned?

**Figure 3:**
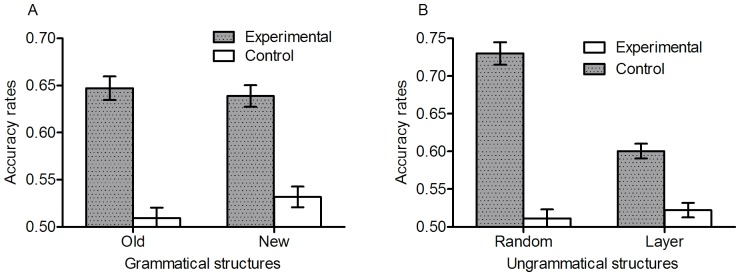
Accuracy rates for old vs. new-grammatical sequences and random- vs. layer-ungrammatical structures under inclusion.

Figure 3 shows mean accuracy rates for old vs. new grammatical structure and random- vs. layer-ungrammatical structures. Table 1 shows mean accuracy rates organised according to the old-/new-grammatical distinction (novelty) or the violation type (layer violations vs. random) under inclusion and exclusion instructions for each group. The difference between inclusion and exclusion will be analyzed below, as will the difference between the different grammars. The analyses in this section are on just the inclusion items, pooling over different types of grammar. Firstly, to examine whether people can generalize the knowledge they acquired in the training to new grammatical structures, we divided the performance for test pairs into performance for old and new grammatical items. A mixed model ANOVA on accuracy rates with grammatical (new vs. old) as a within-participant variable and training (trained vs. control) as a between-participants variable revealed that overall the trained participants classified more accurately than the control participants, *F*(1, 159) = 88.83, *p*<.001, η*_p_* ^2^ = .36, indicating that training result in learning something about the structure of the grammars. It is important to note here that the range of the results (between 50 and 70%) may appear low with respect to traditional (explicit) learning measures. For experiments exploring unconscious, implicit knowledge, these results are relatively high (cf [1], [18]). There was no main effect of old versus new, *F*(1, 153) = .51, *p* = .48; further, for just the trained participants, there was no difference detected between new and old items, *t*(79) = .57, *p* = .57. Old versus new did not interact with any effect of interest, so we will not explicitly consider this factor further.

**Table 1:** Accuracy Rates for Grammatical and Ungrammatical Structures under Inclusion and Exclusion in Each Group.

			Trained				Control			
			Grammatical		Ungrammatical		Grammatical		Ungrammatical	
			Old	New	Layer	Random	Old	New	Layer	Random
Tail embedding	Left-branching	Inclusion	.59(.02)	.62(.02)	.56(.02)	.67(.03)	.52(.03)	.52(.02)	.53(.02)	.50(.02)
		Exclusion	.60(.02)	.60(.02)	.56(.01)	.68(.03)	.51.02)	.54(.02)	.52(.02)	.52(.02)
	Right-branching	Inclusion	.64(.02)	.62(.02)	.59(.02)	.73(.03)	.51(.03)	.55(.02)	.52(.02)	.53(.03)
		Exclusion	.62(.02)	.61(.03)	.59(.02)	.68(.03)	.54(.02)	.55(.02)	.55(.02)	.55(.03)
Centre embedding	Left-branching	Inclusion	.67(.02)	.66(.02)	.63(.02)	.74(.03)	.51(.02)	.55(.03)	.51(.02)	.54(.02)
		Exclusion	.64(.01)	.59(.02)	.57(.02)	.73(.02)	.54(.02)	.59(.02)	.54(.02)	.62(.02)
	Right-branching	Inclusion	.69(.03)	.65(.03)	.62(.03)	.78(.03)	.51(.02)	.51(.02)	.52(.01)	.48(.02)
		Exclusion	.65(.03)	.65(.02)	.62(.02)	.73(.03)	.51(.01)	.53(.02)	.52(.02)	.51(.03)

To explore whether people can detect the specific violations in layer-ungrammatical items as well as the gross violations in random-ungrammatical ones, a mixed model ANOVA with ungrammatical (layer- vs. random-ungrammatical) and training (trained vs. control) as independent variables revealed that overall the trained participants classified more accurately than the control participants, *F* (1, 159) = 114.02, *p*<.001, η*_p_* ^2^ = .42, confirming that learning about the grammatical structure did occur. Further analysis revealed that performance in the trained was greater than that in the control condition for both layer-ungrammatical, *t* (159) = 5.66, *p*<.001, *d* = .90, and random-ungrammatical, *t* (159) = 11.45, *p*<.001, *d* = 1.82, indicating that participants in the trained condition acquired not only broad differences between grammatical and non-grammatical items, as indicated by the sensitivity to random baseline structures, but subtle differences as well, as indicated by the sensitivity to layer violations. We will explore these subtle differences further.

**Figure 4:**
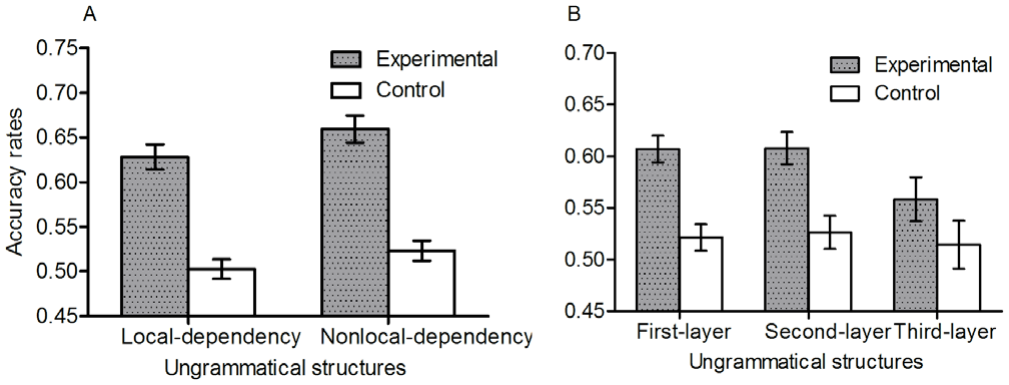
Accuracy rates for different types of structural violation under inclusion.

Figure *4* shows accuracy rates for the different types of structural violation under Inclusion. Trained participants were more accurate than controls for when the violation occurred in each of the first and second layers, *t* (159) = 4.67, *p*<.001, *d* = .74, *t* (159) = 3.67, *p*<.001, *d* = .58, respectively (both significant after Hochberg's, 1988, sequential Bonferroni correction), though not in the third layer. The sensitivity to violations in different layers does not necessarily imply that participants must have parsed the sequence into the embedded parts per se, nor that participants have learnt the long distance dependencies created by the recursive nature of the grammar. Crucially, however, stimuli with layer violations can be divided into local and nonlocal dependency structures based on whether or not the ungrammatical stimulus involves a nonlocal violation (of a nonlocal structure). Trained participants performed better than controls on both local dependencies, *t* (159) = 3.86, *p*<.001, *d* = .61, and nonlocal dependencies, *t* (159) = 5.16, *p*<.001, *d* = .82 (see Table 2). This key result is explored further below. The performance for nonlocal dependencies surprisingly turns out to be higher than for local dependencies. This might be because non-local violations have an intermitting structure and therefore potentially an irregular transition at more than one location (We should not presume that local n-gram structure is always the easiest. In a yet unpublished study, where sequences of letters were explicitly constructed as obeying or violating either global repetition structure or bigrams, participants learnt the repetition structure considerably better than the bigram structure).

**Table 2:** Accuracy Rates for the different types of structures: number of layers (i.e. complexity), the layer where the violation occurred, and dependency type (local vs. nonlocal) under Inclusion.

	Violated layer			Dependency	
	First	Second	Third	local	nonlocal
Experimental	.61(.01)	.61(.02)	.56(.02)	.58(.01)	.65(.02)
Control	.52(.01)	.53(.02)	.51(.02)	.52(.01)	.53(.02)

### Were some types of grammars easier than others?

**Figure 5:**
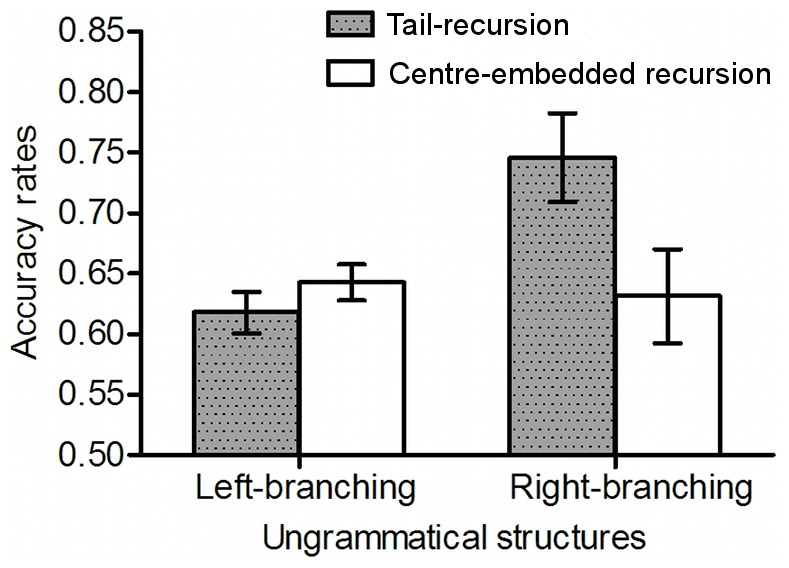
Accuracy rates for nonlocal and local dependencies in the inclusion test, according to type of grammar.

Figure 5 shows the means and standard deviations for proportion of correct classifications of long range dependencies in the inclusion test, according to type of grammar. We subjected the classification of long-distance dependencies in the inclusion test to a 2 (branching: left vs. right)×2 (tail vs. centre embedding) between participants ANOVA. It revealed a significant centre embedding effect, *F* (1, 76) = 4.03, *p*<.05, η*_p_* ^2^ = .05, indicating that the tail embedding grammar was better learned than the centre embedding one. The interaction of centre embedding by branching reached significance, *F* (1, 76) = 5.77, *p*<.05, η*_p_* ^2^ = .07. Further analysis revealed that the tail embedding grammar was better learned than the centre embedding grammar when the branching was left-branching, *t* (38) = 3.37, *p*<.01, *d* = 1.09; and the left-branching was better learned than the right-branching when the grammar was tail embedding, *t* (38) = 2.15, *p*<.05, *d* = .70.

### Was the knowledge conscious or unconscious?

The conscious status of the knowledge can be investigated by the difference between inclusion and exclusion performance [111] and also by the relation of confidence to performance [116]. We consider each method in turn. Although the exclusion instruction was opposite to inclusion instruction, we computed accuracy rates of both inclusion and exclusion performance on the basis of the rate with which grammatical stimuli were chosen.

**Figure 6:**
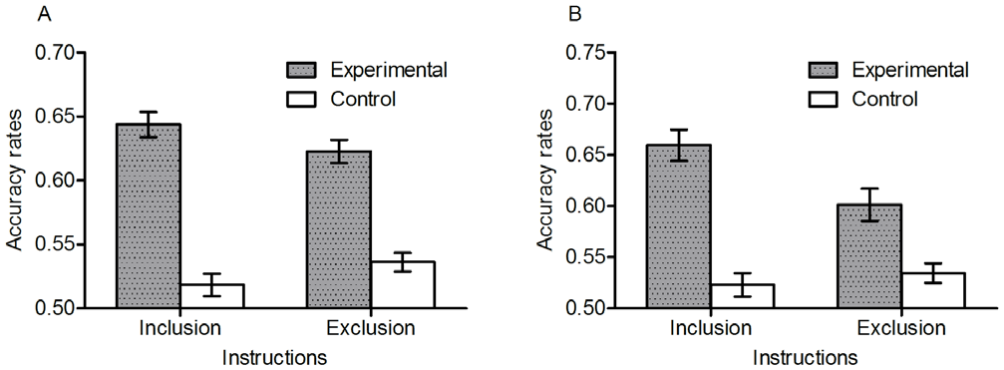
Accuracy rates comparing inclusion and exclusion performance with respect to left- and right-branching grammars.

Figure 6 shows the means and standard errors for proportion correct separated by inclusion and exclusion. An ANOVA on totaracy rates with instruction (inclusion vs. exclusion) a awihin-subject variable and training (trained vs. control), as between-subjects revealed a significant instruction by training interaction, *F* (1, 159) = 8.07, *p*<.01, η*_p_* ^2^ = .05. Overall, trained participants selected more grammatical items in inclusion than exclusion, *t* (79) = 2.60, *p*<.05, *dz* = .29, indicating some control over the use of their knowledge. The effect, though significant, is small. Crucially, exclusion performance was still significantly better than the control group, *t* (159) = 7.46, *p*<.001. *d* = 1.09. That is, when asked to pick the non-grammatical items the trained participants still picked the grammatical items, a result inconsistent with participants consciously knowing that the grammatical items were grammatical.

In terms of the confidence measures of the conscious status of knowledge, when participants said they were purely guessing, inclusion performance was better than that of the control group (*M* = .60, *SE* = .03 vs. *M* = .50, *SE* = .03), *t* (131) = 2.44, *p*<.05, *d* = .43, indicating again that participants were not aware of knowing the grammatical status of the items. When participants had some confidence (>50%), inclusion performance was better than when people said they were guessing (*M* = .66, *SE* = .01 vs *M* = .60, *SE* = .03), *t* (62) = 2.51, *p*<.05, *d* = .64, indicating that people were sometimes aware of knowing an item was grammatical, consistent with the small amount of control that participants exerted. However, when participants had some confidence, the inclusion-exclusion difference was not greater than when participants said they were guessing (*M* = .02, *SE* = .01 vs. *M* = .06, *SE* = .04), *t* (58) = 1.09, *p* = .28, consistent with people not knowing when they had control over the use of their knowledge.

For the classification of long-distance dependencies, a comparable ANOVA revealed a significant instruction by training interaction, *F* (1, 159) = 8.13, *p*<.01, η*_p_* ^2^ = .05. Trained participants selected more grammatical items in inclusion than exclusion, *t* (79) = 2.92, *p*<.01, *dz* = .32, indicating some control over the use of their knowledge. Importantly, exclusion performance was also significantly better than that of the control group, *t* (159) = 3.07, *p*<.01, *d* = .49, indicating that participants did not consciously know that the grammatical items were grammatical. There were only about 14, 18, 12 and 8 long distance dependency trials per subject in the centre and tail embedding left- and branching groups, respectively, so they were not subdivided further into confidence bins. Nonetheless, the exclusion performance demonstrates participants' knowledge of long distance dependencies was largely unconscious.

### What was learnt, controlling for n-grams and repetition structure at word and class levels?

Although the stimuli were generated based on the discussed context-free grammars, participants' performance may not necessarily based on the knowledge of the grammar but on other acquired structures [22], [120]. In particular, we aimed to explore the extent to which participants' responses indicated sensitivity to nonlocal dependencies when other factors were controlled for. For this purpose we employed a (logistic) regression analysis as is common in implicit learning research (e.g. [47], [100], [121]). In order to establish that participants' sensitivity to long distance structure (violations which involved a layer that was intermitted) was not (just) based on knowledge of bigrams or trigrams of words in the training phase we determined for each test item the total (summed) frequency of its bigrams and trigrams of words (word chunk strength) as well as bigrams and trigrams of grammatical classes (class chunk strength) representing grammatical class. Anchor positions (stimulus beginnings and endings) are known to provide important cues which participants pick up [93], [122]. These features were controlled for by the fact that our n-gram analysis coded stimulus beginnings and endings with two different padding symbols, so that anchor positions would be accounted for as potential predictors. We also controlled repetition structure, which can be coded in a number of ways [83]. showed a particularly strong predictor of responding in artificial grammar learning was adjacent repetition structure [123]. Adjacent repetition structure reflects the similarity of a given terminal element to that immediately preceding it, for example, the adjacent repetition structure of AABBCC is 10101. The initial 1 represents the fact that the second letter is the same as the first letter; the following 0 indicates that the third letter is different to the second letter, and so forth. The frequency of the adjacent repetition pattern of words as well as classes of each test item in the training phase was determined. In addition we also controlled for global repetition structure in a similar way. For instance, the item AABBCC has global repetition structure 112233, meaning the first element also appears in the second position, the third position contains a new type of element, which repeats in the fourth, and so on. The two structures ABABC and BCBCD share the identical global repetition structure: viz 12123.

For all test choices and for each participant, the participant's choice (correct/incorrect) was logistically regressed on the difference between the grammatical and non-grammatical items in: Word and class chunk strength, word and class local repetition structure, word and class global repetition structure, as well as a dummy predictor variable which coded whether the violation in the ungrammatical stimulus involved a local (rather than a long-distance) violation (1 for local vs. 0 for long distance). Since local violation was a controlled dummy variable complementary to nonlocal dependencies, the intercept encodes the effect of nonlocal dependencies (see Table 3). Results in the exclusion condition were scored as if they were under inclusion instructions, i.e. “correct” means selecting the grammatical item. The intercept represents the person's ability to classify long distance dependencies with chunk strength, local and global repetition structure controlled on word and class levels, i.e. the intercept is the predicted performance when all these other variables are zero. T-tests over participants showed that participants were not sensitive to word chunk strength as far as we could detect, *t* (79) = 1.11, *p* = .27, but were sensitive to class chunk strength, *t* (79) = 2.30, *p*<.05, *dz* = .26, word local repetition structure, *t* (79) = 3.15, *p*<.01, *dz* = .35, word global repetition structure, *t* (79) = 3.98, *p*<.001, *dz* = .44. Crucially we show that people were sensitive to long distance dependencies with other factors controlled, *t* (79) = 2.43, *p*<.05, *dz* = .27. The latter result is the key result and key reason for performing the analysis: We show learning of long distance dependencies controlling a range of other structures we already know people can implicitly learn. In particular, while global repetition structure is a type of long distance dependency, we show that people are sensitive to the long distance dependencies in the grammar in a way that goes beyond sensitivity to global repetition structure as such. That is, our interpretation of the performance results above in terms of local vs. nonlocal dependencies remains after controlling relevant variables.

**Table 3:** Logistic Regression Analyses Regressing Participants' Responses Applying Surface and Deep-structure Chunk Strength, Local and Global Repetition Structure and Local Dependencies as Predictors.

	Regression coefficient	*p*	*t* (79)
Nonlocal dependency (intercept)	0.150*	0.018	2.43
2–3-grams (word)	0.004	0.270	1.11
2–3-grams (class)	0.002*	0.024	2.30
Local dependencies	0.163	0.071	1.83
Global repetition (word)*	0.032	0.000	3.98
Global repetition (class)	−0.004	0.285	−1.08
Local repetition (word)	0.170*	0.002	3.15
Local repetition (class)	−0.002	0.280	−1.09

* : p<.05.

Note we demonstrate sensitivity to class chunk strength controlling for word chunk strength, a finding that goes beyond the now common demonstration that people are sensitive to chunks of terminal elements, e.g. letters (e.g. [106]; cf [21]). However, the effect size is tiny. The evidence from this analysis therefore that people learnt classes is people's sensitivity to long distance dependencies between word classes controlling for a range of word level variables. Indeed a Bayes factor was run comparing the null hypothesis of no effect to a theory that expected class chunk strength to affect classification by up to 3%. The Bayes factor showed the evidence was 100 times stronger for the null! The predictions of the theory was represented by a half-normal with a standard deviation of 1.5%, i.e. the theory allowed effects between 0 and about 3%; see [124]–[125] for the technique. This is an example of a case where an effect is so tiny that a significant result is actually evidence for the null over a theory predicting a difference as small as is often picked up with abstract implicit learning (cf [18]).

However, the sensitivity to nonlocal dependencies might arise because of sensitivity to nonlocal dependencies between words and not classes. Accordingly the purpose of the next analysis was to examine whether fixed nonlocal chunks (i.e. chunks which would be intermitted) rather than flexible nonlocal dependencies would be potential predictors for participants' responses. The span of intervening material for nonlocal dependencies was 2 or 3 words. Thus we conducted another regression in which bigrams were coded as a) A - - B, where A and B are words and the dashes can be any intervening word: These are long distance word bigrams with two intervening items; and b) A - - - B, long distance word bigrams with three intervening items. If overall sensitivity to long distance dependencies remains, it shows such sensitivity was not based just on learning long distance word bigrams. In the same regression we considered another question. If people are sensitive to the phrase structure, they will be sensitive to phrases as such: Their sensitivity to long distance dependencies will not be for fixed lengths, but to lengths that vary from item to item depending on phrase length. Thus, as a stricter test of people learning long distance dependencies as a consequence of learning hierarchical embedding, we added two more variables to the regression: c) ) A - - B, where A and B are classes and the dashes can be any intervening class: These are long distance class bigrams with two intervening items; and d) A - - - B, long distance class bigrams with three intervening items. Note that c) and d) encode sensitivity to fixed length dependencies; if people have learnt the phrase structure per se, their sensitivity to long distance dependencies will exceed the variance accounted by these fixed-length predictors because there will be variance in when sensitivity is to 2 versus 3 words long not explained by either fixed-length variable. a)–d) could not be added to the regression already performed because there would be too many predictor variables and the regression becomes unstable. Thus we set up a new regression with a) to d) as predictors, as well as a control variable coding whether the item has short distance or long distance dependencies at all. The intercept codes whether there is sensitivity to the long distance dependencies when all these predictors are controlled. Table 4 displays the results. The long distance word bigrams had no predictive power; but long distance class bigrams (for fixed distances) had some. Crucially, the intercept was still significant controlling all of a) to d). Thus, sensitivity to long distance dependencies was not just based on sensitivity to long distance dependencies to words for fixed lengths (2 and 3 intervening items); this is evidence that there was sensitivity to word classes. Further, sensitivity to long distance dependencies was not just based on sensitivity to long distance dependencies to classes for fixed lengths (2 and 3 intervening items); this is consistent with people learning the phrase structure per se. Ideally, one regression would be performed with all predictors in, so our conclusion regarding learning phrase structure per se must await further testing; but we have at least found (unconscious) long distance dependency learning of classes (which goes beyond what has been previously demonstrated).

**Table 4:** Logistic Regression Analyses Regressing Participants' Responses Applying Surface and Deep-structure Nonlocal Chunks as Predictors.

	Regression coefficient	*p*	*t* (79)
Nonlocal dependency (intercept)	0.280*	0.000	5.85
Nonlocal bigram with two intervening (word)	0.003	0.660	0.44
Nonlocal bigram with three intervening (word)	0.009	0.290	1.06
Nonlocal bigram with two intervening (class)	0.006*	0.000	4.49
Nonlocal bigram with three intervening (class)	0.000	0.873	0.16
Local versus Nonlocal	0.019	0.757	0.31

* : p<.05.

### Category identification test

Overall the trained participants could not pick two out of three words of the same category at above baseline levels (baseline being .33; *M* = .31, *SE* = .01), *t* (79) = −1.79, *p* = .084, and similar to the control groups (*M* = .31, *SE* = .01 vs. *M* = .32, *SE* = .01), *t* (159) = −.84, *p* = .40. Further, no group individual was above baseline (all *p*s>.27) and nor was better than their control group (all *p*s>.05). The upper limit of the 95% confidence interval for trained participants is .33, so whatever knowledge trained participants have available for classifying this task, it is not enough to classify more than a percent above chance baseline. The test very sensitively rules out knowledge allowing discrimination of class. Thus, not only were participants not conscious of the grammatical classes they were sensitive to in parsing the structure of sentences, their knowledge of the classes was in such an implicit or embedded form it could not allow first order discrimination of what words had the same class.

## Discussion

The aim of the experiment was to investigate whether participants could implicitly acquire hierarchical recursive structures that resemble natural language word order on an abstract level. A second aim was to further explore the effects of branching-type and centre embedding. Our results showed that trained participants performed much better than the controls with respect to the layer-ungrammatical structures, including long-distance dependencies, under both inclusion and exclusion tests, suggesting that they did implicitly acquire knowledge that enabled them to distinguish the hierarchical structures. Importantly, people's unconscious knowledge of long-distance dependencies goes beyond the now common demonstration that people are sensitive to chunks of terminal elements. Based on the present results we cannot infer which mental representation participants had acquired, however, the findings suggest that it is a form of representation that incorporates long-distance dependencies and likely nested structures. Finally, participants learned better when the grammar featured tail rather than centre embedding (in the sense of [103]–[104], [126]), showing a variable argued to affect preferential learning in natural languages also affects learning of artificial languages in the lab.

### Can people learn recursive structures?

Our approach to determining whether people had acquired distinctively recursively embedded structures was to show that people could become sensitive to the long distance dependencies generated by context-free grammars; and further to show that this sensitivity remained after controlling for n-grams and repetition structure. Thus, in a sentence with multiple levels of recursive embedding, we showed participants could learn to become sensitive to violations of nested embedded structures. As the violation spans the embedding there is suggestive evidence of learning and representing recursively-generated long-distance dependencies. In this respect our results differ from the findings by [77]. While [77] found that people were not able to acquire indexed A^n^B^n^ grammars, the grammar they employed was highly abstract (and requires indexing in order to avoid a counting confound). The grammar used in our study was modelled to be more complex than the simple A^n^B^n^ grammar and to be a more general example of a context-free grammar, with further analogies to some realistic features of constituent order. The variety of structures and greater redundancy of our grammars rather than A^n^B^n^ grammars may in fact render our grammar more learnable than the A^n^B^n^ grammar.

However, although we showed people can discriminate grammaticality, this finding alone does not demonstrate the learning of recursive rules or recursive parsing. We know that people are also sensitive to bigram and trigram frequencies [105]–[107] and repetition structure [82], the latter being a type of long distance dependency which does not need to be recursively specified. To further explore whether participants' knowledge only included bigrams or trigrams of terminal elements or classes in the training phase, we determined for each test item the total (summed) frequency of its bigrams and trigrams of terminal elements (word chunk strength) as well as bigrams and trigrams of grammatical classes (class chunk strength). The performance for long-distance dependencies was significantly above chance when chunk and repetition structure knowledge was controlled for. We suggest the explanation is the acquisition of abstract knowledge at the complexity level of long-distance dependencies and hierarchical, embedded structures. The explanation is admittedly only indirectly supported by the evidence in that we have not decisively shown the psychological reality of the structural hierarchy per se. It should be further stressed that our results do not demand that the stimuli were parsed and processed recursively, as outlined in the introduction. The present training sequences (as all finite sets of sequences) could be entirely represented by a (much less parsimonious) finite-state grammar (encoding every single stimulus) or by mere whole-sequence memorisation. The fact, however, that the set used novel new-grammatical sequences (generated by the context-free rules) plausibly rules out a catch-all finite-state representation or whole-sequence memorisation. The fact that participants performed well for new-grammatical structures (and therefore were generalising) indicates that a more complex explanation for their learning behaviour is required. Accordingly, we controlled for other known alternative explanations of participants' response patterns and that they only account in a limited way for the performance. Nonetheless, if a potential explanation which involves learning and matching recursive structure is right, people trained on some depth of embeddings should be able to generalise to other novel structures and to other levels of embedding, to within the limit of the relevant memory buffer. Further research, for example click experiments or segmentation tasks in which participants are instructed to group word sequences that belong together, should also be able to provide evidence of levels of embedding being psychologically relevant structural units. Our paradigm provides an ideal starting point for such further research as well as neuroscientific research exploring whether the neural pathways involved in processing during this experiment resemble other results based on context-free structures.

Many computational models of implicit learning tend to be good at learning chunks and associations [110]. For example, the SRN is good at learning conditional probabilities of successive elements [127]. Nonetheless it can learn the musical inversions of [99], but only by learning them as long-distance associations [109] rather than as a recursively generated structure per se. How the SRN might cope with learning hierarchically embedded structures, as in the current material, remains to be determined in future work. Chunking models (e.g. [106], [128]–[129]) are challenged by the data because such models assume that learning involves chunking of adjacent elements. For example, the competitive chunk (CC) model assumes that the probability of a letter string is judged grammatical on the basis of the network of chunks acquired during the memorization task. Although the CC model can successfully reproduce some findings with the artificial finite grammar task, it is unlikely to learn the relations between grammatical classes over long distances when bigrams and trigrams are controlled. A further issue to be explored in future research is the impact of left or right branching and potential for centre embedding on model performance.

### Is the learning unconscious?

In order to demonstrate that people implicitly learnt the grammars, we need to show people acquired unconscious knowledge [116]. We employed both the PDP method and confidence ratings to determine people's awareness of knowing the grammaticality of items. As applied to this experiment, PDP is based on the assumption that if one consciously knows whether or not an item has the same structure as the training items, one should be able to control whether the item is endorsed as familiar or unfamiliar. Confidence ratings directly measure whether one consciously knows whether or not an item has the same structure as the training items. Both methods indicated substantial amounts of unconscious knowledge and some, but very limited, conscious knowledge. Specifically, people were quite likely to pick the grammatical item (and reject the non-grammatical) when deliberately trying to pick the item which violated the structure of the training items; and when people thought they were guessing and trying to pick the well structured item, they tended to pick the grammatical item (and reject the non-grammatical). These conclusions apply overall and for non-grammatical items violating only long distance dependencies. The finding that the learning outcome is in fact implicit (contrast e.g. [50]) is important since it demonstrates that the implicit learning mechanism can develop sensitivity to structures that are beyond mere chunks. Importantly, explicit learning of context-free structures would be less surprising since, for instance, the learning of a programming language involves dealing with an artificial language of this type of grammar. Thus testing for awareness of the learned structures is crucial in this context.

### Did participants acquire word classes?

An important contribution of the study is in providing evidence for the implicit learning of relations over classes. People were apparently processing more than just mere surface based features of the stimulus sentences and were able to infer some knowledge about syntactic categories from the surface word sequences in an unsupervised manner. The fact that they, on the other hand, were at chance at the word class tests at the end of each experiment shows that they could not directly or consciously access their knowledge of these classes even though their response patterns indicated they applied it. This finding takes previous findings that people can learn word classes in artificial grammar learning experiments [102] one step further by showing such knowledge can be unconscious.

### Is the learning affected by the type of grammar?

Natural grammars differ in a number of structural ways, some of which have been argued to affect their ease of learning (e.g. [103]–[104], [126]). One such structural feature is the extent to which the grammar produces centre embedded clauses. The less centre embedding the grammar has the potential to produce, the easier the grammar should be learn and use. On the other hand, from a theoretical perspective there is no structural difference between left and right branching per se in terms of complexity. Consistently and as one would expect, participants trained on the tail rather than centre embedding grammars performed better, which accords with our predictions. But to be more precise, participants performed best with the left-branching tail-embedding grammar than any of the other grammars. Strikingly, the participants' own native (left-branching) grammar is centre embedding, yet the easiest grammar was one with tail-embedding, suggesting that fundamental cognitive factors could override extensive experience with a centre embedding grammar.

From a psycho-linguistic perspective, the findings regarding the performance interactions with branching type and centre embedding link with linguistic findings. The performance advantage for left-branching structures probably reflects the fact that their native language is left branching – a fact to be further explored with participants of different native language in future work. The advantage of tail versus centre embedding grammatical structures is, on a basic level, strongly related to Hawkins's performance and correspondence hypothesis for natural grammars [104]. Typological studies find a preference towards tail embedding (or “consistent”) structures in languages of the world (cf [130]) and this difference seems to accord with our findings that in both, the left- and right-branching cases the structures which do not feature centre-embedding are better learned than the centre embedding ones. This seems to suggest a potential performative or structural effect that impacts on the learning of syntactic word order and might ultimately constitute a driving force in the way how grammars are selected or evolve (cf [104], [131]).

The very fact that structural properties affected the learning of our artificial grammars in explicable ways given general cognitive constraints and the participant's native grammar supports our contention that the hierarchical structures of our grammars were learnt as such. This fact also illustrates how issues in linguistics can both motivate and be explored by the use of artificial grammars.

## Conclusion

Overall, our findings suggest that people can implicitly acquire knowledge of tail- and centre-embedding structures (involving long-distance dependencies) as well as word classes drawn from recursive context-free grammars in the lab that are similar at an abstract level to those in natural language. The knowledge includes relations between grammatical classes even for dependencies over long distances, in ways that go beyond simple relations (e.g. n-grams) between individual words. Even though in real world, the interaction between syntax and semantics affects and facilitates the parsing as well as the acquisition of language, the finding of learning and processing of such hierarchical dependencies on a structural level is an important contribution. Our study shows how such complex forms of word sequences are potentially acquired incidentally from exposure and represented implicitly, i.e. based on unconscious knowledge. Notably, incidental learning is not the same as implicit learning; while reading this paper you incidentally acquired much conscious knowledge, e.g. on roughly which pages were certain points made. Implicit learning is the acquisition of unconscious knowledge, which can occur both incidentally and intentionally [117]. For an example of implicit learning that is intentional, the dynamic control tasks of Berry and Broadbent provide an example (see, for instance, [132]). Second language learning potentially provides another example of intentional implicit learning, albeit a (more) controversial one (cf. [14], [15]). While explicit knowledge of complex context-free languages (such as explicitly acquired knowledge of programming languages like ML or C) is less surprising, showing the ability that participants incidentally acquire implicit knowledge of context-free languages is novel and relates to natural acquisition processes like language or music acquisition (e.g. [14], [15], [8]). We further found that the differences in grammatical complexity between tail- and centre-embedding and right or left branching affect the learning performance. This accords with Hawkins's performance and correspondence hypothesis for natural grammars and provides a hint towards a cognitive preference for adjacent tail- rather than centre-embedding structures which afford some ease of processing.

## Supporting Information

Appendix S1 **Finite-state representations of formal grammars used in two studies** [**34]**, [85] **.**
(DOC)

Appendix S2 **Stimulus materials used in the study.**
(XLS)
