# Supplementary material for: Implicit Learning of Recursive Context-Free Grammars
Source: PLoS One. 2012 Oct 19;7(10):e45885. doi: 10.1371/journal.pone.0045885 (PMC3477156; doi:10.1371/journal.pone.0045885)
Supplement: Appendix S1 — Finite-state representations of formal grammars used in two studies [34] , [85] . (DOC) [file pone.0045885.s001.doc]

**Appendix 1**

Three grammars used in other studies can be expressed by regular / finite state grammars:

*(1) Language P* (Saffran, 2002, [34])

S → AP BP (CP)

AP → A (D)

BP → CP F

CP → C (G)

Through iterative substitution of the nonterminals AP, BP and CP, a regular expression is obtained:

S → A (D) C (G) F C (G)

*(2) Language N:* (Saffran, 2002, [34])

S → AP BP

AP → [(A) | (D)] (must have at

least one; if both, A precedes D)

BP → CP F

CP → [(C) | (G)] (must have at least one; if both, C precedes G)

regular expression:

S → [A | D | AD] [C | G | CG] F

The grammar used by Saffran (2001, [85]):

S → AP BP (CP)

AP → A (D)

BP → CP F | E

CP → C (G)

regular expression:

S → A (D) [C (G) F | E] C (G)
